# Development of Natural Polysaccharide–Based Nanoparticles of Berberine to Enhance Oral Bioavailability: Formulation, Optimization, Ex Vivo, and In Vivo Assessment

**DOI:** 10.3390/polym13213833

**Published:** 2021-11-05

**Authors:** Kanchan Kohli, Ali Mujtaba, Rozina Malik, Saima Amin, Md Sarfaraz Alam, Abuzer Ali, Md. Abul Barkat, Mohammad Javed Ansari

**Affiliations:** 1 Department of Pharmaceutics, School of Pharmaceutical Education & Research, Jamia Hamdard, New Delhi 110062, India; ali_jh82@yahoo.com (R.M.); samin@jamiahamdard.ac.in (S.A.); 2 Departments of Pharmaceutics, Faculty of Pharmacy, Northern Border University, Rafha 73213, Saudi Arabia; 3 Department of Pharmaceutics, College of Pharmacy, Jazan University, Jazan 88723, Saudi Arabia; sarfarazpharma1981@gmail.com; 4 Department of Pharmacognosy, College of Pharmacy, Taif University, P.O. Box 11099, Taif 21944, Saudi Arabia; abuali@tu.edu.sa; 5 Department of Pharmaceutics, College of Pharmacy, University of Hafr Al-Batin, Al Jamiah, Hafr Al-Batin 39524, Saudi Arabia; abulbarkat05@gmail.com; 6 Department of Pharmaceutics, College of Pharmacy, Prince Sattam Bin Abdulaziz University, AlKharj 11942, Saudi Arabia; javedpharma@gmail.com

**Keywords:** berberine, alginate, chitosan, nanoparticles, pharmacokinetic study

## Abstract

The phytogenous alkaloid berberine (BBR) has become a potential drug for the treatment of diabetes, hyperlipidemia, and cancer. However, its therapeutic potential is limited because ofpoor intestinal absorption due to its efflux by the *P*-gp expressed in the intestinal lumen. Therefore, we aimed to design and fabricate a nanoparticulate system for delivery of BBR employing naturally derived biodegradable and biocompatible polymers, mainly chitosan and alginate, to enhance the oral bioavailability of BBR. A chitosan-alginate nanoparticle system loaded with BBR (BNPs) was formulated by ionic gelation method and was optimized by employing a three-factor, three-level Box-Behnken statistical design. BNPs were characterized for various physicochemical properties, ex vivo, and in vivo evaluations. The optimized BNPs were found to be 202.2 ± 4.9 nm in size, with 0.236 ± 0.02 of polydispersity index, zeta potential −14.8 ± 1.1 mV, and entrapment efficiency of 85.69 ± 2.6%. BNPs showed amorphous nature with no prominent peak in differential scanning calorimetry (DSC) investigation. Similarly, fourier-transform infrared spectroscopy (FTIR) studies did not reveal any interaction between BBR and excipients used. The drug release followed Higuchi kinetics, since these plots demonstrated the highest linearity (R^2^ = 0.9636), and the mechanism of release was determined to be anomalous or non-Fickian in nature. An ex-vivo gut permeation study showed that BNPs were better internalized into the cells and more highly permeated through the intestine. Furthermore, in vivo pharmacokinetic analysis in female Wistar rats showed a 4.10−fold increase in the oral bioavailability of BBR from BNPs as compared to BBR suspension. With these findings, we have gained new insight into the effective delivery of poorly soluble and permeable drugs via a chitosan-alginate nanoparticle system to improve the therapeutic performance of an oral nanomedicine.

## 1. Introduction

Berberine chloride (BBR) is a naturally derived isoquinoline alkaloid found in several medicinal herbs, namely, *Berberis aristata, Coptis japonica*, *Coptis chinensis*, *Hydrastis canadensis*, *Phellondendron amurense,* and *PhellondendronchinenseSchneid* [1]. It has been primarily recommended for diarrhea and gastroenteritis, including many other biological activities, such as hypoglycemic, hypolipidemic, antiproliferative, antineoplastic, and antiarrhythmic activities [2]. It has also been found effective against diabetes, metabolic syndrome, hypertension, and polycystic ovary [3]. Thus, the drug has appeared as a therapeutic agent against multispectral activities. Due to a large number of biological activities, low cost, and low toxicity, BBR has gained more interest. BBR is prepared by chemical synthesis and available in the market as chloride and in sulfate-salt form. It is available as conventional tablets or capsules forms either alone or in combination. BBR is a yellowish crystalline powder with a bitter taste that is either odorless or has a slight odor. It is sparingly soluble in methanol and slightly soluble in ethanol and water. However, its salt forms are somewhat more soluble [4].

The wide application of BBR is, however, greatly limited by its poor intestinal absorption. The oral bioavailability of drugs depends primarily on the rate and extent of their dissolution from its dosage form. The absolute bioavailability of BBR following oral administration in rats has been reported to be below 1% [5,6]. The low bioavailability of BBR is attributed to its poor aqueous solubility and dissolution, as well as its permeation by glycoprotein (*P*-gp) through multidrug efflux pumps [7,8,9].

The most preferred route of drug administration is oral delivery, especially to treat chronic diseases. Many drugs fail to meet their needed therapeutic action due to their poor bioavailability, which is mainly due to poor solubility, low permeability, and/or high metabolism. Nanoparticle drug delivery systems have been extensively studied worldwide to improve the oral bioavailability of drugs with poor aqueous solubility, particularly those belonging to BCS class II and IV [10,11]. Biopolymers have been used as basic components of nanosystems. Specifically, chitosan and alginate have high availability, a nontoxic nature, an ease of handling, and low cost, making these polymers special and attractive choices as building blocks for nanoparticles [12].

The current research work was aimed to develop polymeric nanoparticles of BBR using alginate and chitosan as biopolymers, both of which are *P*-gp inhibitors. Further, alginate and chitosan are known to be biocompatible, biodegradable, mucoadhesive, and permeation enhancers. These properties, together with the small size of the particles, help them to cross the biological barriers and are sufficient to increase the bioavailability of BBR, which was achieved remarkably and successfully. The effect of primarily independent variables (concentration of chitosan, sodium alginate, and calcium chloride) affecting the dependent variables (particle size, polydispersity index, and zeta potential) was analyzed using a Box–Behnken design (BBD). Furthermore, the optimized formulation was characterized for various physicochemical properties inex vivoandin vivoevaluation.

## 2. Materials and Methods

### 2.1. Materials

BBR was procured from Kisalaya Herbals, Indore, India. Polymers and chitosan (medium molecular weight; degree of acetylation, 85%) were purchased from Sigma, Cedex, France, and sodium alginate (molecular weight between 75–100 kDa) was purchased from S.D Fine Chemicals, New Delhi, India. For analysis, HPLC-grade water was used, which was taken from E-Merck India Ltd., Mumbai, India. All other chemicals and reagents, unless otherwise mentioned, were of analytical grade.

### 2.2. Preparation of Berberine-Loaded Nanoparticles (BNPs)

BNPs were prepared by cation-induced controlled gelation of sodium alginate [13]. The alginate solution was made by dissolving the polymer in distilled water and adjusted the pH with hydrochloric acid (1M HCl) to 5.0–5.3. Chitosan solution was prepared in 1% glacial acetic acid solution. For the pregelation step, 10 mL of calcium chloride solution was added to alginate solution in drop−by−drop with constant stirring on a magnetic stirrer at 700 rpm for 30 min. BBR (5mg) was dissolved in 1 mL of dimethylsulfoxide (DMSO), and it was loaded at the pregelation step. Then, 20 mL of chitosan solution was applied dropwise to the pregel step to form self-assembled nanoparticles (NPs). In order to increase overall uniformity and disperse particles, the NPs solution was stirred for 60 additional minutes, then sonicated for 15 min, and finally homogenized for 30 min at 22,360 g forces and allowed to equilibrate for 24 h. An ultrafiltration membrane was used, the NPs were washed with water three times to remove extra BBR and unreacted substances on the surface.

The schematic representation for the preparation of nanoparticles is as follows:

- 10 mL solution of calcium chloride was added dropwise to the 50 mL alginate solution with stirring (Pregelation step).
- At pregelation step: added BBR (5 mg was dissolved in 1 mL of DMSO)
- Kept it on magnetic stirrer for 30 min
- Gelation step: added 20 mL of chitosan solution drop by drop
- Continue stirring for 60 min
- Sonicated for 15 min
- Finally homogenized for 30 min at 22,360 g-forces
- Kept the formulation for 24 h

Different BNP formulations were prepared and optimized using three-factor, three-level BBD using Design Expert^®^ software (Version 10, State-Ease Inc., Minneapolis, MN, USA). Chitosan (X_1_), sodium alginate (X_2_), and calcium chloride (X_3_) were selected as independent variables, with their low (−1), medium (0), and high levels (+1) used for the preparation of 17 formulations. Particle size (PS) (Y_1_), polydispersity index (PDI) (Y_2_), and zeta potential (ZP) (Y_3_) were chosen as dependent variables (Table 1).

### 2.3. HPLC Method of Analysis

For the analytical study, an HPLC system with LC-10AT VP pump, SPD-M10A VP photodiode array detector (Shimadzu, Japan), a Rheodyne injection valve fitted with a 20 μL injection loop, and CLASS-VP (Version 6.12 SP5, Tokyo, Japan) integration software were used. Baseline resolution was obtained using a Phenomenex Luna C-18 column (Phenomenex Inc., CA, USA) at 25 ± 2 °C. The mobile phase was passed via a PVDF filter of 0.45 μm, which was degassed before use. The flow rate was maintained constant at 0.6 mL/min, and detection was performed at a wavelength of 270 nm [14]. For the calibration plot, different concentrations of BBR (5, 7.5, 12.5, 17.5, and 22.5 μg/mL) were prepared using water as the solvent. The standard drug peak was compared with the peak of the drug sample. The HPLC calibration curve was plotted in water and plasma for determination of entrapment efficiency, drug release, and pharmacokinetics in rat plasma, respectively.

### 2.4. Characterization of BNPs

#### 2.4.1. PS, PDI, and ZP Measurement 

PS, PDI, and ZP assessment was performed through zeta sizer with DLS technique (Nano-ZS, Malvern Instruments, Malvern, UK) and analyzed by ‘DTS Nano’ program. The prepared BNP formulations were diluted with Milli Q water and shaken vigorously to get an intensity of around 100–250 kV S^−1^. The measurement of PS, PDI, and ZP was performed for all formulations.

#### 2.4.2. Determination of Entrapment Efficiency (EE) and Drug-Loading (DL) Efficiency 

The EE and DL potential of the BNP formulation was measured by isolating drug-loaded NPs from the aqueous phase containing non-associated BBR using ultracentrifugation (REMI Corporation, India) for 30 min at 20,000 rpm. 1mg of BNPs was taken and then dissolved in 1 mL of DMSO solution, a common solvent for BBR and alginate-chitosan complex, to dissolve them completely [15]. The above HPLC method was used to analyze the prepared solution, and the EE and DL were calculated using the given equations:

(1) $$\%\mathrm{EE}=\frac{W1-W2}{W1}\times 100$$

(2) $$\%\mathrm{DL}=\frac{W1-W2}{W3}\times 100$$

where *W_1_* = amount of BBR loaded in the NPs, *W_2_* = amount of non-entrapped BBR, and *W_3_* = weight of NPs

### 2.5. Differential Scanning Calorimetry Analysis

The physical state of BBR in pure-sample drug, physical mixture, and BNPs was analyzed with (Perkin-Elmer Pyres 6 DSC, Massachusetts, USA). The samples (2 mg) were weighed individually and put into an aluminum pan and then closed. As a reference standard, an empty, closed aluminum pan was placed on the alternate side. The thermal scanning was done at the rate of 10 °C/min, between 40 and 300 °C. The thermogram was recorded using Pyris™ software under nitrogen gas at a flow rate of 20 mL/min.

### 2.6. FTIR Spectroscopy Analysis

FTIR spectroscopic (Shimadzu, Kyoto, Japan) studies were performed with the KBr pellet technique. BBR and BNPs (about 5 mg) were separately mixed with KBr (100 mg) and were ground into a fine powder and compressed into a pellet under a hydraulic pressure at 10,000 psi. The FTIR spectra were recorded for each sample at a wave number ranging from 400 to 4000 cm^−1^ using Win-IR software.

### 2.7. Microscopic Evaluation of BNPs

Analysis of NPs’ structural size and surface morphology was carried out using transmission electron microscopy (TEM) and scanning electron microscopy (SEM) [16]. The surface topography of optimized BNPs was observed with SEM (EVO LS 10 Zeiss, Carl Zeiss Inc., Oberkochen, Germany). The sample was spread on a slide and kept for drying. After drying, the sample was placed on carbon tape and coated with conductive gold-palladium under an argon environment in a high-vacuum evaporator using a gold-sputter module. The coated samples of NPs were then scanned at an accelerating voltage of 13.52 kV, and photomicrographs were taken. Using smart SEM program tools, data analysis was performed. The surface morphology of BNPs was analyzed with Philips CM 100 TEM. The samples were processed on copper grids, negatively stained with uranyl acetate (2%), and air-dried before analysis. The samples were analyzed in TEM (JEOL JEM1010, Tokyo, Japan) at 100 kV accelerated voltage, and data collection was performed on the AMT image–capture engine.

### 2.8. In Vitro Drug-Release Study

The in vitro dissolution tests were performed to analyze the drug release from BNPs formulations. The release study was carried out with a dialysis membrane bag (10 kDa molecular cutoff) in phosphate buffer pH 7.4 with 0.5% *v*/*v* tween 80 dissolution media. The membrane was soaked overnight in the dissolution media for activation before use. A total of 10 mg of freeze-dried BNPs was dissolved, and the equivalent quantity of BBR suspension was put in the dialysis bag and placed in dissolution media, which were stirred continuously at 150 rpm for 24 h at 37 ± 0.5 °C. The samples were withdrawn at predetermined time intervals, filtered using a 0.45μm syringe filter, and analyzed through the above-mentioned HPLC method. An equal volume of fresh dissolution media was replaced to ensure proper sink condition.

### 2.9. Stability Studies

Based on the guidelines given by ICH Q1A (R2), the stability studies of final optimized BNPs were carried out. The BNP samples were stored for 3 months in a stability chamber (TH 90S, Thermolab, Mumbai, India) at 5 ± 2 °C and 25 ± 2 °C. The samples were analyzed for physical appearance, size analysis, drug content, and total percentage drug release at 0, 30, 60 and 90-day intervals [17].

### 2.10. Ex Vivo Gut-Permeation Study 

Ex vivo gut permeation study of BBR and BNP formulations was performed using a non-everted rat gut sac model [18,19]. The protocol for the gut permeation experiments was approved by the Institutional Animal Ethical Care Committee (IAEC) of Jamia Hamdard, India (173/CPCSEA/2012). Wistar rats of 180–200 g had fasted for 12 h before euthanasia with free access to water and were housed under standard settings. The animal was sacrificed, and the ileum part of the small intestine was cut, sliced longitudinally. A 5 cm long ileum part was cut and rinsed with Krebs solution to remove feces and blood.

In the gut sac, BBR and BNPs equivalent to 10 mg of BBR were loaded. The sac was then put in the jacketed glass, having 10 mL of Krebs solution, which was pre-heated to 37 ± 0.5 °C, and oxygenated via an aerator for 120 min. The sample was removed at a specific time interval and filtered and analyzed by the above-mentioned HPLC method for the drug content. The quantity and %drug permeation from the sac were determined and compared.

### 2.11. In Vivo Pharmacokinetic Study

Pharmacokinetic analysis was performed for optimized BNP formulations and plain BBR suspension to check the improvement of bioavailability of BBR, as per the approved experimental protocol no. 173/CPCSEA/2012 from the IAEC, Jamia Hamdard, New Delhi, India. The experiment was carried out in female Wistar rats with 180–200 g bodyweight that were kept in a cage under standard conditions (55 ± 5%RH and 25 ± 2 °C) with free access to a standard diet (Lipton feed, Mumbai, India) and drinking water. The animals were divided into three groups (*n* = 6), in which group I acts as a control and was given normal saline orally. Group II was given BBR suspension, and group III received the BNP formulation at a dose of 100 mg/kg orally. Blood samples were taken at various times after oral administration, and the plasma was separated by centrifugation at 2500× *g* for 20 minutes at 4 °C and kept at −20 °C until further examination. For bioanalysis of the sample, it is required to put the sample at room temperature, and BBR was isolated using protein precipitation technique with acetonitrile. The samples were then centrifuged, and the supernatant was collected, filtered, and analyzed by the HPLC method (Shimadzu LC-10 VP, Tokyo, Japan). Addins-PK Solver and Pk1Pk2 program for Microsoft Excel were used to determine the various pharmacokinetic parameters of BBR, such as maximum plasma-drug concentration (C_max_), the time to reach maximum plasma-drug concentration (T_max_), and area under curve AUC_0__→t_. The relative bioavailability of the formulation was also calculated with a plain drug suspension. The pharmacokinetic results were statistically evaluated using the GraphPad Prism program (Instat 3.06, Software Inc., San Diego, CA, USA), and using the student’s *t*-test, statistical differences were measured.

## 3. Results and Discussion

### 3.1. Formation and Characterization of BNPs

The chitosan-alginate nanoparticles of BBR were prepared by the ionotropic gelation method, which is a simple, rapid, and reliable method. The emulsification and organic-solvents stages were not included in the ionotropic gelation process, thereby reducing the inactivation of encapsulated drugs [20]. In the case of NPs, the addition of chitosan into the calcium-alginate pregel containing the BBR solution and the calcium alginate pregel simultaneously traps the BBR solution suspended in the medium. It is suggested that NPs can be formed by enveloping the negatively charged calcium alginate complex in a pregel state with chitosan–cationic polymer, and the pregel state is required to allow the ionic interactions between calcium, alginate, and chitosan to form NPs [21].

To statistically optimize the formulation variables, the BBD was used [22]. In order to establish the required conditions for the inclusion of the hydrophobic drug BBR into the chitosan-alginate NPs, several experiments had to be performed. A group of 17 experiments (BNP1–BNP17) was carried out for three factors at three levels each for the response surface methodology comprising BBD. In Table 1, the observed responses for all 17 formulations are shown with their independent variables of experimental runs. The amine groups of chitosan are protonated, and the carboxyl groups of alginate are ionized in aqueous solutions at a pH 5.2, which is the most important for the formation of the polyionic complex [23]. The hydrogel is also formed at pH 5.2 by amino-group interaction with carboxylate groups of alginate. The positively charged BBR was initially complexed with negatively charged alginate to obtain a maximum drug loading to the NPs.

The variation in PS, PDI, and ZP of the BNPs was dependent on the ratio of chitosan %, alginate %, and CaCl_2_% solution. By varying chitosan, alginate, and CaCl_2_ concentrations, there was a huge variation in particle size as well. The reason could be that a particular concentration of each polymer reacts with the other polymers and the rest of the amount could adhere to the surface of another polymer, hence increasing the size and causing irregularity in the shape of particles as well. The BNPs were best optimized with a stirring time of 30 min for pregelation and 60 min for gelation. Similar work was done previously, and the results obtained were almost the same [24]. All the results obtained were successfully correlated with the BBD.

**Figure 1:**
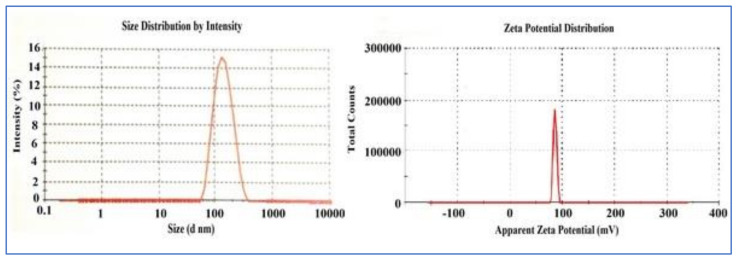
(**a**) Particle size and (**b**) Zeta potential of BNPs (mV).

The design expert software’s point prediction was used to evaluate the optimum formulation of BNPs (0.04% *w*/*v* chitosan concentration, 0.5% *w*/*v* alginate, and 0.1% *w*/*v* calcium chloride) that predicted optimized process parameters to be 219.5 nm for PS, 0.291 for PDI, and −15.1 mV for ZP. The optimized BNP formulation was prepared, and the observed mean PS was 202.2 ± 4.9 nm, with a PDI of 0.236 ± 0.02 and ZP of -14.8 ± 1.1 mV, as shown in Figure 1a,b, respectively (*n* = 3). The predicted values of optimized formulation were compared with their experimental values for all responses, with their percentage prediction error as shown in Table 2. The results for the PS, PDI, and ZP were found within the limits, and the calculated (%) prediction error ensures the reliability of the developed equations, which reveals the applicability of the RSM model [25].

### 3.2. BBR loading and Entrapment in BNPs

The EE and DL of BBR from optimized BNP formulations were found to be 85.69 ± 2.6% and 8.55 ± 0.23%, respectively. The entrapment of BBR in BNPs was a great challenge, and the pH of the alginate and chitosan solution played an important role in the EE and DL in the chitosan-alginate complex. Different pH was tried, but ultimately, the entrapment efficiency and loading capacity were best optimized with the pH of chitosan at 5.3 and that of alginate at 5.6. Similar results were also deduced by others [26]. The entrapment and loading efficiency were also dependent on the stirring time and speed of the formulation. When the stirring speed and time were increased, the entrapment and loading of the drug in BNPs were decreased, which was most probably due to the leaching of the drug due to collision among the particles and with the intact surfaces of the container for a longer time.

### 3.3. DSC Analysis

**Figure 2:**
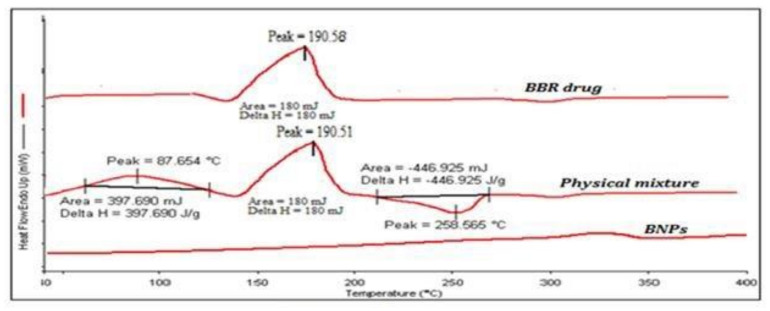
DSC thermograms of BBR, physical mixture, and BNPs.

The melting point (m.p.) of BBR was found very close to the reference when analyzed on DSC and was found to be 191.58 °C [9]. In the physical mixture of BBR, chitosan, and alginate, the m.p. peak of BBR was found to be 190.51 °C, which showed no type of interaction between the excipient polymers and the drug. However, the DSC thermogram of BNPs revealed the absence of a BBR peak. The reason for the absence of a BBR peak in BNPs could be most probably due to the entrapment of BBR in solution form, or the drug could be in amorphous form. Figure 2 shows the DSC thermogram of BBR, the physical mixture of BBR with chitosan and alginate, and BPNs.

### 3.4. FTIR Spectroscopy Analysis

**Figure 3:**
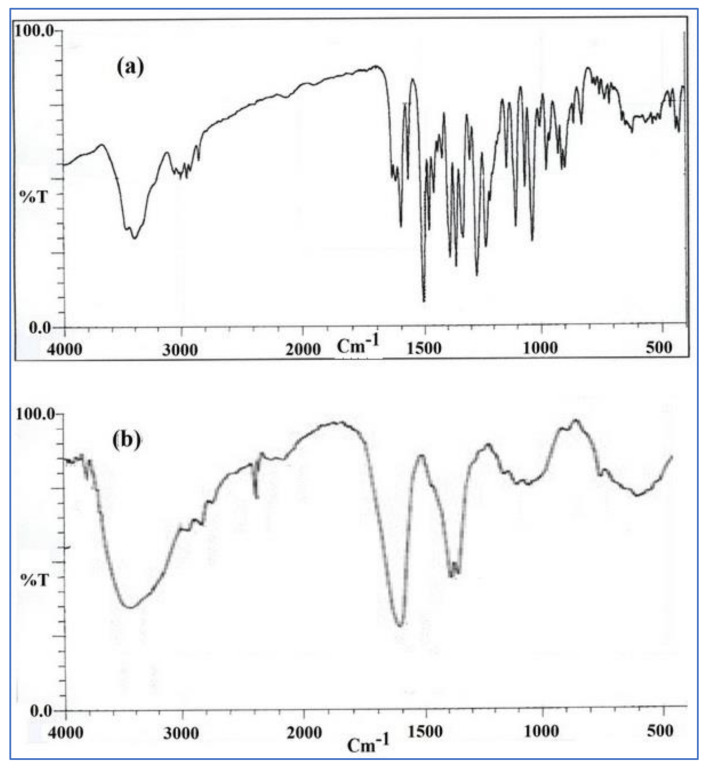
FTIR spectra of (**a**) pure BBR and (**b**) BNPs.

The presence of a methoxyl group (peak at 2.844 cm^−1^) was revealed by the FTIR spectrum of BNPs, and the peak at 1.635 cm^−1^ is assumed to correspond to the iminium (C=N^+^) double bond present in the molecule. In addition, the 1569 and 1506 cm^−1^ signals reflect the aromatic bending of C=C and the furyl group, respectively [27]. Similar peaks are there in the BBR, which clearly support the results of DSC and reveal that there was no chemical interaction among the BBR, chitosan, and alginate in BNPs. Figure 3a,b show FTIR images of standard drug BBR and BNPs.

### 3.5. Nanoparticle Morphology

**Figure 4:**
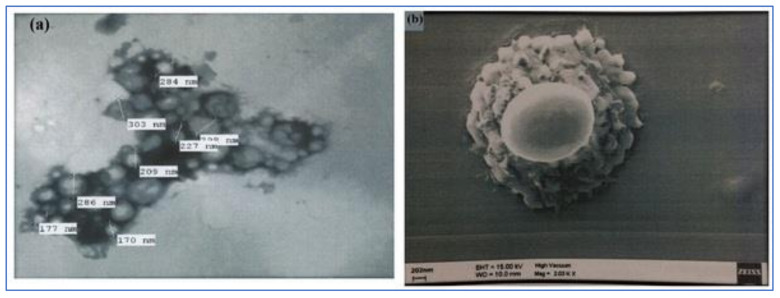
**(a**) TEM image of BNPs (**b**) SEM image of BNPs.

The combination of SEM and TEM is the direct method to evaluate the size, surface morphology and vesicular structure of NPs [19]. The TEM image of BNPs showed the discrete particles with a solid, dense structure, with a size ranging from 170 to 303 nm, which was in agreement with the results obtained from the PS measurement by the DLS technique (Figure 4a). The surface morphology of BNPs was analyzed by SEM techniques, and the SEM image results showed an irregularly smooth and rough surface (Figure 4b).

### 3.6. In Vitro Drug-Release Kinetics

**Figure 5:**
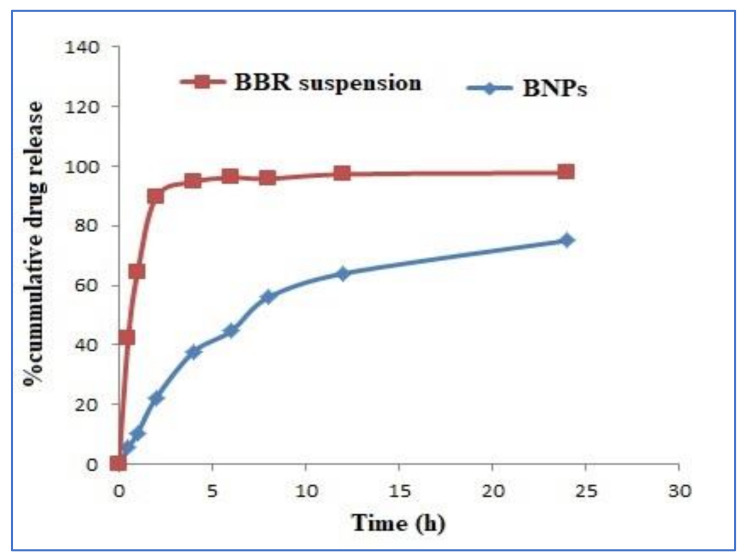
Cumulative release of BBR from BBR suspension and BNPs.

The release study was conducted on lyophilized BNPs and was compared to that of free BBR suspension. It was observed that 100% of the free BBR was released in about 4 h, whereas optimized BNP formulation showed an initial burst release of 10.49 ± 1.5%, followed by a continuous and controlled release of 75.06 ± 4.2% of BBR in 24 h from BNPs (Figure 5). This initial release from BNPs could be most probably because of the presence of the drug on the surface of NPs or due to the diffusion of the drug from the pores, which are nearer to the surface of NPs [28]. Since NPs have a larger surface-area-to-volume ratio, such geometry is also favored for fast release [29].

To determine the release order, the in vitro release profile of the BNPs was kinetically analyzed. In the various release-kinetic models, such as zero-order, first-order, Higuchi, Hixcon-Crowell, and Korsmeyer-Peppas models, the data of the drug released were used, and the best-fit model was selected based on the value of regression coefficient (R^2^) [29]. It can be seen in Table 3 that BBR from BNPs follows the Higuchi release, for which a maximum R^2^ value of 0.9636 was observed, which revealed the drug release from a matrix-diffusion-controlled system. The Korsmeyer–Peppas semi-empirical model was used to analyze the drug-release behavior from BNPs formulation on the linear proportion of the curve. The exponent constant (*n*) was found at 0.39, which suggested an anomalous Fickian-diffusion release, which reveals the drug release could be by both diffusion and erosion mechanisms.

### 3.7. Stability Studies

The optimized formulation of BNPs was evaluated for physical appearance, size analysis, drug content, and total %drug release at various time intervals. It was observed that the BNP formulation showed no significant change in physical appearance, a very small change in mean PS, which was possibly due to aggregation of NPs over a period [11], and very little or no change in drug content in the three-month study period (Table 4). At both temperature conditions, i.e., 5 ± 2 °C and 25 ± 2 °C, no significant changes were seen in the total BBR released from BNPs.

### 3.8. Ex Vivo Gut-Permeation Study

**Figure 6:**
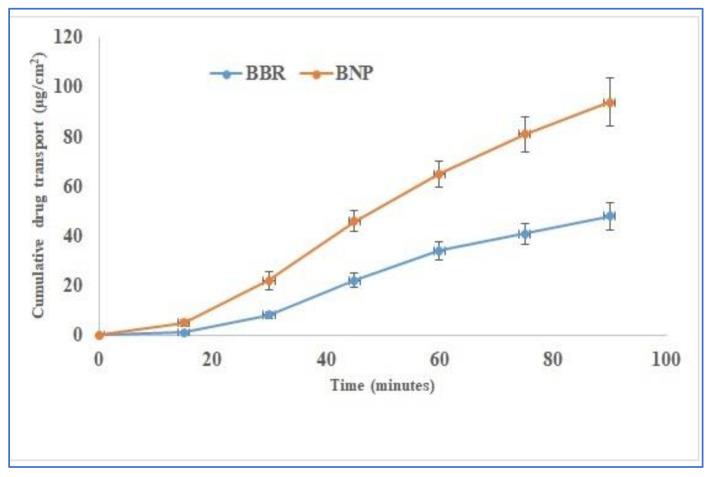
Ex vivo gut-permeation study showing comparative cumulative drug transport.

Figure 6 represents the intestinal permeation profiles of BBR suspension and BNPs. It is evident from the graph that the permeation of BBR is enhanced 1.95–fold higher in BNPs than in BBR suspension. The enhancement of BBR permeation in BNPs could be most probably due to the inhibition of *P*-gp’s expression on the gut lumen by the known *P*-gp inhibitors, i.e., chitosan and alginate [30]. The enhancement in permeation can also be attributed to the entrapment of BBR in nanoparticles, which otherwise is eliminated from the body, having only 5% bioavailability [31]. Similarly, NPs themselves have a small size and also play an important role in enhancing permeation through the intestine [32,33,34].

### 3.9. Pharmacokinetic Studies

**Figure 7:**
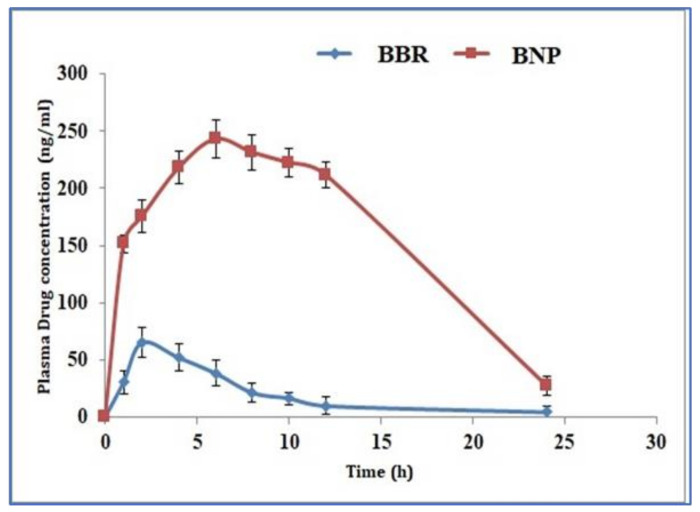
In vivo pharmacokinetics profile of BBR after oral administration of BBR suspension and BNPs (mean ± SD, *n* = 6).

The in vivo performance of the BBR suspension and BNPs was assessed for their pharmacokinetic performance for further verification of the above experiments and proof of concept. Pharmacokinetic analysis was carried out using the non–compartmental (model–independent) method [18,19]. Figure 7 shows the plasma concentration-time profiles of BBR from BBR suspension and BNPs after oral administration, while Table 5 shows the various pharmacokinetic parameters.

It was found that the C_max_ of BBR from BNPs (230.57 ± 8.30 ng/mL) significantly increased by 3.41-fold (*p* < 0.05) as compared to BBR suspension (67.54 ± 3.90 ng/mL). This significant increase in C_max_ value may be due to the *P*-gp inhibition by alginate and chitosan polymers, which are present in BNPs that enhance BBR absorption from the enterocytes. The T_max_ value of BNPs was also found to be significantly higher than BBR suspension, which may be due to sustained release of BBR from BNPs.

The areas under the curve (AUC_0–24_) that denote the extent of absorption for BBR suspension and BNPs were found 910.87 ± 28.30 ng·h/mL and 3758.14 ± 199.89 ng.h/mL, respectively. This means that the BBR absorption from BNPs was significantly higher than BBR suspension (*p* < 0.05). The pharmacokinetic data revealed that the BNPs have better oral bioavailability, as compared to BBR suspension, by a factor of 4.13. This could be due to the *P*-gp inhibition by chitosan and alginate, which can prevent the drug efflux from the enterocytes [30]. Hence, these findings also support the gut-permeation study. The previously published work showed that the bioavailability of BBR in anhydrous reverse micelle and polymer-lipid hybrid nanoparticles was enhanced 2.4–fold and 3.34–fold only compared to BBR solutions [35,36]. Hence, our BNP formulations have good absorption efficiency and can enhance the medical performance of BBR as compared to existing formulations.

## 4. Conclusions

The oral formulation of poorly bioavailable drugs is challenging and needs further investigation. Developing an oral formulation for drugs like BBR with bioavailability <5% is very challenging for pharmaceutical scientists. BNPs were successfully prepared by the ionic gelation method using *P*-gp inhibitors chitosan and sodium alginate, which could minimize the efflux of the drug out of the enterocytes. This also facilitated the entry of the drug through the intestinal cells. The size of the optimized BNPs was found to be 202.2 ± 4.9 nm, with 0.211 ± 0.02 of PDI. FTIR and DSC analyses suggested the there is no chemical interaction between BBR and the major excipients (chitosan and alginate) of the formulation. The SEM and TEM images of optimized BNPs showed smooth, spherical NPs with well-defined boundaries. The drug release from BNPs was found to show a burst release followed by a slow release, and it follows an anomalous Fickian-diffusion release, which reveals the drug release could be by both diffusion and erosion mechanisms. In addition, the in vivo pharmacokinetic analysis showed that the BNPs increased 4.10-fold in improving the BBR oral bioavailability. Hence, this developed formulation with amalgamation benefit of the nano-sized system and polymeric *P*-gp modulator was found to be a good replacement for conventional oral formulation with a decrease dose and frequency of dosing.

## Figures and Tables

**Table 1:** Variables and observed responses in BBD.

Formulation Code	Factors Combinations at Different Levels			Response Variables		
	X_1_ = Chitosan (% *w*/*v*)	X_2_= Sodium Alginate (% *w*/*v*)	X_3_ = Calcium Chloride (% *w*/*v*)	Y_1_ = Particle Size (nm)	Y_2_ = Polydispersity Index (%)	Y_3_ = Zeta Potential (mV)
BNP1	0.02	0.3	0.1	248.3 ± 0.02	0.541 ± 0.02	−20.8 ± 2.3
BNP2	0.04	0.3	0.1	270 ± 3.3	0.414 ± 0.05	−20.9 ± 2.3
BNP3	0.06	0.3	0.1	314 ± 6.5	0.621 ± 0.03	−25.4 ± 2.6
BNP4	0.05	0.3	0.1	314.2 ± 9.9	0.568 ± 0.01	−13.6 ± 3.1
BNP5	0.08	0.3	0.1	341.2 ± 2.3	0.761 ± 0.08	−30.8 ± 2.3
BNP6	0.02	0.3	0.2	278.2 ± 4.6	0.496 ± 0.06	−21.8 ± 2.7
BNP7	0.04	0.3	0.2	341.2 ± 5.7	0.521 ± 0.05	−22.8 ± 2.9
BNP8	0.02	0.1	0.1	347.5 ± 4.8	0.376 ± 0.04	−27.8 ± 1.6
BNP9	0.02	0.2	0.1	246.1 ± 5.3	0.295 ± 0.07	−25.8 ± 1.9
BNP10	0.02	0.4	0.1	323.3 ± 6.2	0.504 ± 0.03	−19.8 ± 2.1
BNP11	0.02	0.5	0.1	260.4 ± 2.9	0.491 ± 0.02	−18.4 ± 1.6
BNP12	0.02	0.6	0.1	361.5 ± 4.2	0.594 ± 0.04	−30.8 ± 1.8
BNP13	0.10	0.5	0.1	290.2 ± 8.2	0.435 ± 0.03	−18.6 ± 3.0
BNP14	0.04	0.5	0.1	220.52 ± 3.1	0.311 ± 0.01	−15.1 ± 1.6
BNP15	0.08	0.5	0.1	218.6 ± 5.1	0.672 ± 0.012	−16.1 ± 1.8
BNP16	0.06	0.5	0.1	334.1 ± 2.9	0.446 ± 0.06	−11.9 ± 2.9
BNP17	0.02	0.5	0.1	253.8 ± 6.1	0.582 ± 0.02	−12.7 ± 3.5

**Table 2:** Composition of optimum checkpoint formulations, the predicted and experimental values of response variables, and percentage prediction error.

Optimized BNPs Formulation Composition (X_1_:X_2_:X_3_)	Response Variable	Experimental Value	Predicted Value	Percentage Prediction Error
0.04:0.5:0.1	Y_1_	202.2	209.5	−2.272
	Y_2_	0.236	0.241	−2.075
	Y_3_	−14.8	−15.1	−1.987

**Table 3:** Regression coefficient (R^2^), release exponent (n) from the kinetic equations.

Formulation	Zero-Order		First Order		Higuchi		Korsmeyer–Peppas	
	R^2^	k	R^2^	k	R^2^	k	n	R^2^
BNPs	0.8050	3.113	0.9232	0.0258	0.9636	17.290	0.3907	0.9554
BBR suspension	0.283	2.372	0.4017	0.0535	0.5667	17.045	0.2401	0.6767

**Table 4:** Stability data.

BNPs Formulation (5 ± 2 °C)			
Time (Days)	Change in Physical Appearance	Particle Size ± SD nm	Drug Content
0 30 60 90	No No No No	202.2 ± 1.60 203.5 ± 1.56 203.9 ± 3.21 205.9 ± 1.95	85.54 ± 2.10 84.89 ± 1.46 83.76 ± 1.67 83.23 ± 1.69
**BNPs formulation (25 ± 2 °C)**			
0 30 60 90	No No No No	202.2 ± 1.60 203.7 ± 2.32 205.6 ± 3.29 206.4 ± 3.11	85.54 ± 2.10 84.23 ± 1.26 83.53 ± 1.37 82.98 ± 1.83

**Table 5:** Pharmacokinetic parameters.

Pharmacokinetic Parameters	BBR Suspension	BNPs
C_max_(ng/mL)	67.54 ± 3.90	230.57 ± 8.30
T_max_(h)	2.00 ± 0.12	6.00 ± 0.02
AUC_0ߝ24_ (ng·h/mL)	910.87 ± 28.30	3758.14 ± 199.89
Kel (h^−1^)	0.12 ± 0.01	0.08 ± 0.003
t_1/2_ (h)	5.42 ± 0.99	9.04 ± 0.17
Bioavailability enhancement (F)	-	4.13

## Data Availability

All data generated or analyzed during this study are included in this published article.
